# Patient-Reported Outcome Measures and Decision Regret After Salvage Radical Prostatectomy for Recurrent Prostate Cancer Following Radiotherapy or Focal Therapy

**DOI:** 10.3390/cancers17030396

**Published:** 2025-01-25

**Authors:** Fabian Falkenbach, Johanna Hagemann, Francesca Ambrosini, Pierre I. Karakiewicz, Zhe Tian, Yamini Nagaraj, Burkhard Beyer, Philipp Mandel, Felix Preisser, Derya Tilki, Tobias Maurer, Lars Budäus, Hans Heinzer, Alexander Haese, Thomas Steuber, Georg Salomon, Markus Graefen

**Affiliations:** 1 Martini-Klinik Prostate Cancer Center, University Medical Center Hamburg-Eppendorf, 20246 Hamburg, Germanybudaeus@uke.de (L.B.); heinzer@uke.de (H.H.);; 2 Cancer Prognostics and Health Outcomes Unit, Division of Urology, University of Montreal Health Center, Montreal, QC H2X 0A9, Canada; 3 IRCCS Ospedale Policlinico San Martino, 16132 Genova, Italy; 4 Center for Urological Rehabilitation, Klinik Wildetal, 34537 Bad Wildungen, Germany; 5 Department of Urology, University Medical Center Hamburg-Eppendorf, 20246 Hamburg, Germany

**Keywords:** prostatic neoplasms, quality of life, salvage radical prostatectomy, biochemical recurrence, patient-reported outcome measures, radiotherapy, focal therapy

## Abstract

Background/Objectives: Radical prostatectomy (RP) may be considered for recurrent prostate cancer (PCa) following primary curative-intended local therapy. The effect of different prior therapies on patient-reported outcome measures (PROMs) after RP is not well defined. Methods: Validated PROMs (SF-12, EPIC-26, Decision Regret Scale) were used to compare health-related quality of life (HRQOL) and functional status changes following salvage RP after radiotherapy (RT-sRP) or focal therapy (FT-sRP), relative to primary RP. Results: Among 26,515 RP patients who underwent RP between 2014 and 2024, 107 (0.4%) previously received radiotherapy (RT-sRP) and 98 (0.4%) previously received focal therapy (FT-sRP). Compared with primary patients before RP, only the sexual function of RT-sRP patients was lower (EPIC score, 51 vs. 75, *p* < 0.001). One year after RP, RT-sRP patients exhibited lower functional status in all EPIC-26 domains compared to primary RP patients, whereas FT-sRP patients did not differ significantly. For instance, the median 1 yr EPIC-26 urinary incontinence scores were 46 (RT-sRP), 86 (FT-sRP), and 92 (primary RP). In adjusted mixed model analyses, the detrimental effects of RT-sRP vs. primary RP were further validated. In contrast, no such association was observed for FT-sRP. Decision regret and severe complications were low. Conclusions: Prior FT had only a marginal effect on HRQOL and functional status following RP, while urinary continence and sexual function were lower for RT-sRP patients as compared to primary RP patients. However, from an overall PROM perspective, prior therapies did not exert a prohibitive effect that would preclude RP as a treatment option in those patients.

## Simple Summary

This study compared patient-reported outcomes after salvage radical prostatectomy following radiotherapy or focal therapy with outcomes following primary radical prostatectomy in prostate cancer patients. One year after radical prostatectomy, patients with prior radiotherapy had lower functional status in all domains compared to primary radical prostatectomy, while patients after focal therapy did not differ significantly. Decision regret and severe complications were low for both salvage groups. Prior focal therapy did not compromise the patient-reported outcomes, while prior radiotherapy led to reduced urinary continence and sexual function.

## 1. Introduction

The optimal treatment modality for local recurrence after initial prostate cancer (PCa) treatment is a matter of debate [1,2,3]. Although well-designed clinical trials are lacking, potential treatments include promising but still experimental reirradiation, PSA-based monitoring for low-risk disease, or surgical interventions [1,4,5,6]. Salvage radical prostatectomy (sRP) can provide excellent cancer control outcomes in carefully selected patients at experienced centers [7,8,9,10,11,12,13,14,15]. However, it is recommended rather permissively and frequently underutilized due to concerns about high treatment toxicity and low functional outcomes [1,12,16,17,18,19]. The advent of less morbid initial PCa treatments, such as focal therapy (FT) using high-intensity focused ultrasound (HIFU), questions the generalizability of this assessment on sRP [9,11]. However, the effects of different initial PCa therapies on standardized patient-reported outcome measures (PROMs) following sRP have not been well described. Prior analyses have suggested that sRP toxicity strongly depends on the initial PCa therapy [13,20,21,22]; however, reports relying on standardized PROMs in that realm are scarce [14]. We hypothesized that the detrimental effects of sRP on PROMs would be less pronounced after initial FT than after initial RT. We tested this hypothesis in a contemporary cohort of sRP patients.

## 2. Materials and Methods

### 2.1. Patient Cohort

We identified all consecutive patients who underwent radical prostatectomy (RP) for nonmetastatic prostate cancer (PCa) between 2014 and 2024 within our prospectively maintained institutional database at the Martini-Klinik Prostate Cancer Center, Hamburg, Germany. Salvage RP (sRP) was defined as RP for biopsy-proven local recurrence after initial radiotherapy (RT) or focal therapy (FT). Initial RT was administered either percutaneously or via brachytherapy. All types of initial FT were included (HIFU, irreversible electroporation (IRE), hyperthermia ablation, photodynamic therapy, cryoablation, and other procedures). All RP were performed in an open retropubic or robot-assisted fashion as described before [23]. Neurovascular structure-adjacent frozen-section examination (Neuro-SAFE) was routinely carried out for nerve-sparing [24]. All patients consented to the procedure, follow-up, and data analysis. This retrospective analysis was approved by the local review board.

### 2.2. Patient-Reported Outcome Measures

Validated patient-reported outcome measures (PROMs) were collected prior to RP, then at one and six months following RP, and annually thereafter, as previously described [25]. The validated 12-item Short Form Health Survey (SF-12) measured general health-related quality of life (HRQOL), subdivided into physical and mental scores [26,27,28]. The disease-specific functional impact of RP was measured using the validated 26-item Expanded Prostate Index Composite (EPIC-26) with the following domain-specific function summary scores: urinary incontinence, urinary irritative, sexual, hormonal, and bowel. Changes in each EPIC-26 domain were considered clinically meaningful if the validated minimally important differences (MID) were met: urinary incontinence 6–9, urinary irritative 5–7, sexual 10–12, hormonal 4–6, and bowel 4–6 [29]. Higher EPIC-26 or SF-12 scores indicate better function and HRQOL. Distress or remorse was measured using the 5-item Decision Regret Scale (DRS) [30,31]. A higher DRS represents increased regret. PROMs were collected using the International Consortium for Health Outcomes Measurement-certified [32] Questlink^®^ platform (Koninklijke Philips N.V., Amsterdam, The Netherlands) in English [33] or in a validated Norwegian or German translation [34,35]. Full and social continence were defined as no or ≤1 (safety) pads per day, respectively. Potency was defined as the ability to maintain spontaneous erection sufficient for penetrative intercourse. Complications were classified according to Clavien–Dindo (perioperative or during follow-up) [36]. The questionnaire data were not censored for subsequent treatments. The number of non-missing items required for each summary score were as following: SF-12 12, EPIC-26 urinary incontinence 4, EPIC-26 urinary irritative 4, EPIC-26 bowel 5, EPIC-26 sexual 5, EPIC-26 hormonal 4, and DRS 4.

### 2.3. Statistical Analyses

Differences were assessed using Pearson chi-square (categorically coded variables) or Wilcoxon rank-sum (continuously coded variables) tests. Primary RP patients were defined as the reference group to assess the additional toxicity of prior therapies. Linear mixed-effects models (R packages: lme4, lmerTest) adjusted to patient age at RP, surgical approach (open vs. robot-assisted), cancer characteristics at RP (Gleason grade group and pT-stage), and year of surgery were used to identify potential correlations between prior PCa therapy (FT, RT, primary) and PROMs after RP repeatedly measured over time at the patient level (applying a conditional growth model with the random slope removed). All statistical analyses were performed using R version 4.4.1 (14 June 2024) [37]. All tests were two-sided, with the level of significance set at *p* < 0.05.

## 3. Results

### 3.1. Patient and Surgery Characteristics

Among 26,515 RP patients who underwent RP between 2014 and 2024, 107 (0.4%) previously received radiotherapy (RT-sRP) and 98 (0.4%) previously received focal therapy (FT-sRP) (Table 1). PROMs were available for 23,426/26,515 (88.4%) patients at a median (IQR) follow-up period of 3 (1, 5) years. Among the RT-sRP patients, RT was administered percutaneously in 69/107 (64.5%) and as brachytherapy in 38/107 (35.5%) patients. Among these, low dose rate (LDR) and high dose rate (HDR) brachytherapy was previously performed in 29/38 (76.3%) and 6/38 (15.8%; 3 NA) patients, respectively. HIFU was initially used in 50/98 (51.0%) FT-sRP patients, followed by IRE (20/98, 20.4%), hyperthermia ablation (16/98, 16.3%), photodynamic therapy (5/98, 5.1%), cryoablation (3/98, 3.1%), and other procedures (4/98, 4.1%). Between the initial PCa diagnosis and RP, a median of 44, 23, and 3 months elapsed for RT-sRP, FT-sRP, and primary RP patients, respectively. Compared to their primary counterparts, RT-sRP patients were older (median, 67 vs. 64 years, *p* < 0.01) and exhibited more frequently adverse cancer characteristics at RP such as Gleason grade group 4–5 (31.8 vs. 9.6%), ≥pT3-stage (63.6 vs. 40.9%), or pN1 (29.0 vs. 13.2%, all *p* < 0.001). Conversely, no such differences were observed between FT-sRP and primary RP patients (all *p* > 0.05). In both salvage RP groups, the robot-assisted approach (13.1 or 40.8 vs. 58.0%) was less frequently used compared to primary RP patients. RT-sRP patients exhibited less frequently bilateral nerve-sparing (26.2 vs. 73.6%, *p* < 0.001). Clavien–Dindo > IIIa° complications were observed in 18/107 (16.8%, RT-sRP), 7/98 (7.1%, FT-sRP), and 1717/26,310 (6.5%, primary RP) patients. In RT-sRP patients, these consisted of 8 ureteral strictures, 3 lymphoceles, 2 bowel injuries, 2 hematuria cases, 2 thrombembolic events, and 1 case of postoperative bleeding. In FT-sRP patients, these consisted of 4 hernia cases, 2 ureteral strictures, and 1 subarachnoid hemorrhage.

### 3.2. Health-Related Quality of Life (SF-12)

**Figure 1:**
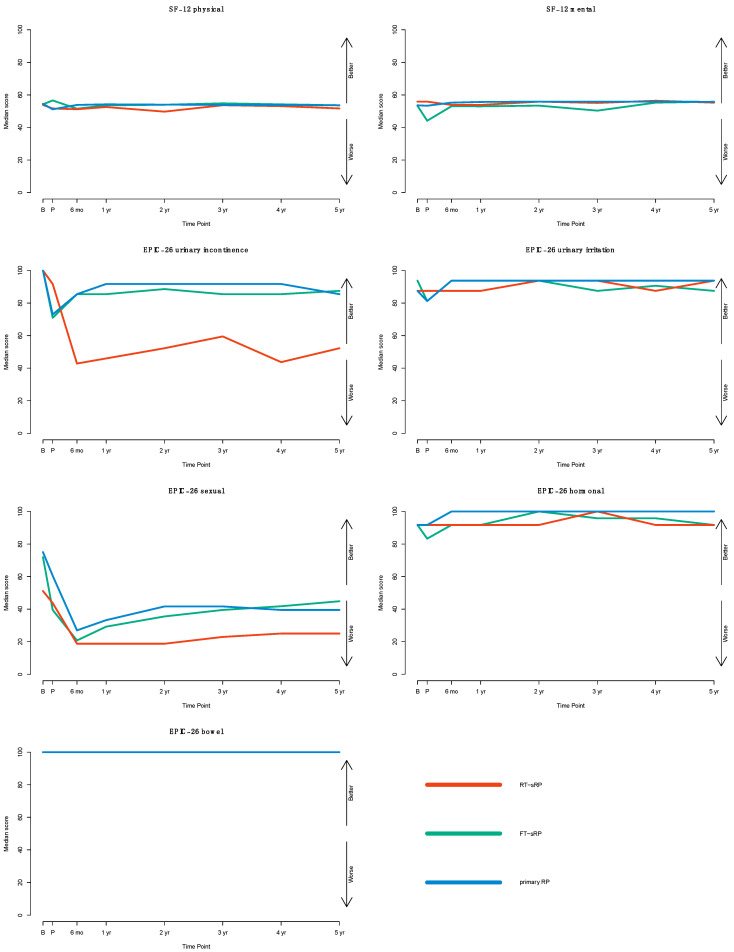
Patient-reported outcome measures of patients undergoing salvage radical prostatectomy following prior focal therapy (FT-sRP, green) or radiotherapy (RT-sRP, red) compared to primary radical prostatectomy patients (blue) between 2014 and 2024 (*n* = 26,515). The time-point is measured relative to the RP procedure. B = one month before RP, *p* = one month after RP, mo = month, yr = year. The solid line represents the median score of each group. The color-coded transparent area shows the mid-interquartile range. Higher scores indicate better quality of life and functional status.

Before RP, no significant differences between RT-sRP or FT-sRP patients were observed in comparison with primary RP patients for SF-12 physical (median, all 54) or mental (56 or 53 vs. 54, Table 2). During follow-up, no significant differences emerged between RT-sRP, FT-sRP, and primary RP (Figure 1). In mixed model analysis (MMA) adjusted to age, surgical approach, cancer characteristics, and year of surgery, RT-sRP or FT-sRP status were no significant predictor of decreased SF-12 physical or mental (all *p* > 0.05).

### 3.3. Impacts on Functional Domains (EPIC-26)

For the EPIC-26 urinary incontinence domain, no significant differences between RT-sRP or FT-sRP patients compared to primary RP patients were observed before RP (median, all 100). In all groups, the urinary incontinence function initially decreased after RP and partly recovered thereafter (Figure 1). One year after RP, RT-sRP patients exhibited a statistically significant and clinically meaningful lower urinary incontinence function score than primary RP patients (46 vs. 92, *p* < 0.001). No such difference was observed for FT-sRP patients (Table 2). Correspondingly, the social continence rates after one year were significantly lower in RT-sRP patients (32/56; 57.1%) than in primary RP patients (12,774/13,750; 92.9%; *p* < 0.001). Conversely, FT-sRP patients did not exhibit lower social continence rates (55/57, 96.5% vs. 12,774/13,750, 92.9%; *p* = 0.4). In MMA adjusted to age, surgical approach, cancer characteristics, and year of surgery, RT-sRP status was a significant predictor of reduced continence over time (*p* < 0.001), whereas FT-sRP was not (*p* = 0.8). In an additional sensitivity analysis, the magnitude of this effect was three times larger for patients after brachytherapy than after percutaneous RT (linear estimate: −17 vs. −5).

For the EPIC-26 urinary irritation domain, no significant differences between RT-sRP or FT-sRP patients compared to primary RP patients were observed before RP (median, 88 or 94 vs. 88). In all groups, the urinary irritation function initially decreased after RP and recovered to baseline levels or higher (Figure 1). One year after RP, RT-sRP patients exhibited a statistically significant and clinically meaningful lower urinary irritation function score than primary RP patients (88 vs. 94, *p* < 0.001). No such difference was observed for FT-sRP patients (Table 2). In MMA adjusted to age, surgical approach, cancer characteristics, and year of surgery, RT-sRP status was a significant predictor of lower irritation scores over time (*p* < 0.01), whereas FT-sRP was not.

Before RP, the EPIC-26 sexual function score was significantly lower in RT-sRP patients compared to primary RP patients (median, 51 vs. 75, *p* < 0.001). Correspondingly, 22/66 (33.3%, RT-sRP) vs. 10,703/16,925 (63.2%, primary) patients were able to maintain a spontaneous erection sufficient for intercourse prior to RP (*p* < 0.001). In all three groups, sexual function initially decreased after RP and partially recovered thereafter (Figure 1). One year after RP, RT-sRP patients exhibited a statistically significant and clinically meaningful lower sexual function score than primary RP patients (19 vs. 33, *p* < 0.001). For FT-sRP patients, no such difference was observed at baseline or after one year (Table 2). Only one RT-sRP patient (of 54 patients who answered this question, 1.9%) reported an erection firm enough for intercourse one year later. In MMA adjusted to age, surgical approach, cancer characteristics, and year of surgery, RT-sRP status was a significant predictor of reduced sexual function (*p* < 0.001), whereas FT-sRP was not. However, in an additional sensitivity analysis relying on MMA also adjusted for nerve-sparing status and the parameters mentioned above, neither RT-sRP nor FT-sRP status were predictors of adverse sexual function (*p* > 0.1).

For the EPIC-26 hormonal domain, no significant differences between RT-sRP or FT-sRP patients compared to primary RP patients were observed before RP (median, all 92). Hormonal function remained high in all groups, and there were no relevant changes for each group during follow-up (Figure 1). One year after RP, RT-sRP and FT-sRP patients exhibited a statistically significant and clinically meaningful lower hormonal function score than primary RP patients (92 and 92 vs. 100, *p* < 0.01). Again, in MMA adjusted to age, surgical approach, cancer characteristics, and year of surgery, RT-sRP and FT-sRP status were no significant predictors of hormonal function (*p* > 0.1).

For the EPIC-26 bowel domain, no clinically meaningful differences between RT-sRP or FT-sRP patients compared to primary RP patients were observed before RP (median, all 100) or one year after (median, all 100). Bowel function remained high in all groups, and there were no relevant changes for each group during follow-up (Figure 1). In MMA adjusted to age, surgical approach, cancer characteristics, and year of surgery, RT-sRP and FT-sRP status were no significant predictors of bowel function (*p* > 0.4).

### 3.4. Decision Regret Scale (DRS)

After one year, the median (IQR) Decision Regret Scale (DRS) scores were 10 (0, 25) for RT-sRP, 10 (0, 25) for FT-sRP, and 0 (0, 20) for primary RP. There were no significant differences between RT-sRP or FT-sRP vs. primary RP patients (all *p* > 0.05).

## 4. Discussion

In the current analysis, less morbid initial PCa therapies resulted in less pronounced detrimental effects on functional status after sRP. We are the first to describe the effect of different prior therapies on standardized and validated SF-12/EPIC-26 questionnaires, while also relying on one of the largest contemporary sRP series. Therefore, the current analysis revealed several noteworthy observations that might counterbalance the current rather permissive (under-)utilization of sRP in general.

First, prior RT had a statistically significant and clinically meaningful detrimental effect on urinary continence after RP, whereas prior FT did not. One year after RP, the median EPIC-26 incontinence function score was 46 for RT-sRP vs. 92 for primary RP (*p* < 0.001). Correspondingly, the 1 yr pad-free continence rates (without safety pad) were 26.8% (RT-sRP), 73.7% (FT-sRP), and 67.6% (primary RP). While no previous report has examined the effect of sRP on standardized PROMs in a similar comprehensive fashion, the differences between full/social continence rates align well with the current findings [9,13,15,19,38,39,40,41,42,43]. For instance, Ribeiro et al. reported 1 yr pad-free rates of 49% after RT-sRP and 83% after FT-sRP [20]. Similar pad-free rates of 39.2% for RT-sRP and 77.3% for FT-sRP were reported by Onol et al. [21]. Conversely, Rodler et al. reported lower and indifferent continence rates of 48.4% (RT-sRP) and 52.9% (FT-sRP, *p* = 0.8) in a German cohort [14]. In systematic reviews, continence rates ranged from 21 to 90% following RT-sRP [8,44] or even from 10 to 100% following sRP after any primary therapy [22]. In contrast, a tendency towards higher and less heterogeneous continence results was observed in FT-sRP studies. For instance, Marconi et al. reported a continence rate of 83% and Herrera-Caceres et al. reported a continence rate of more than 90% following FT-sRP [11,41]. In a systematic review of FT-sRP patients, full continence was achieved in an average of 56.7% of patients [43]. Validating these differences between RT-sRP and FT-sRP also from a pathophysiological perspective, RT may cause more frequently ischemic sphincter damage by vessel obliteration and endarteritis than FT [45]. In a recent systematic review, the average urinary continence rate was estimated to be 67% for FT-sRP vs. 55% for RT-sRP patients [22]. Of note, these excellent results in FT-sRP patients were achieved despite substantial higher rates of prior transurethral resection of the prostate in FT-sRP patients (8.2 vs. 2.0%, *p* < 0.001). In conclusion, not all sRP procedures are identical, and primary PCa therapy has major implications for urinary incontinence after sRP. Detailed assessment of primary PCa therapy is mandatory for individualized patient counseling, and other salvage therapies should be considered as well [12]. Moreover, our results are reassuring for men requiring sRP after FT. This validates the concept of FT itself, because sRP can be offered safely later if further treatment intensification after the initial FT is required.

Second, sexual function declined after RP within the salvage setting, but also after primary RP. In each group, the EPIC-26 sexual function score more than halved one year after RP, compared to pre-RP values. In contrast to previous studies [11,41,42,43], FT-sRP status was not associated with lower rates of nerve-sparing and did not exert a detrimental effect on sexual function compared with primary RP patients (*p* > 0.05). Although we refute the notion that all patients have no erectile function after sRP, RT-sRP are especially vulnerable to these changes because they start with lower sexual function prior to sRP (median, 51 vs. 75, *p* < 0.001) and exhibit higher rates of RP without any nerve-sparing (60.7 vs. 6.6%, *p* < 0.001). In fact, after adjusting for nerve-sparing status in MMA, the detrimental effect of RT-sRP status disappeared. Low rates of nerve-sparing are well documented for RT-sRP patients and often originate from periprostatic fibrosis caused by RT, which renders the correct identification of the intrafascial plane for nerve-sparing challenging [14,15,16,17,20,21]. These poor rates of overall sexual function post RT-sRP are in agreement with previously reported potency rates ranging from 11 to 55%, depending on the applied definition and surgical technique [9,20,21]. Of note, sexual function continued to improve up to three years after sRP in the current analysis, as previously also described for primary RP [46,47,48]. Moreover, it must be considered that sexual function deterioration due to subsequent PCa treatments and aging may artificially inflate the effects of the RP itself. In fact, active surveillance patients have also reported a gradual decline in sexual function over time [49,50,51]. In the current analysis, RT-sRP patients, who were affected most by sexual function decline, had more frequent lymph node invasion at RP (29.0 vs. 13.2%, *p* < 0.001), which is associated with further PCa treatments, and were significantly older (67 vs. 64 years, *p* < 0.01). Taken together, lower sexual function at baseline, lower rates of nerve-sparing, and natural deterioration by aging put the observed detrimental effect of RT-sRP into perspective and limited the overall pertinence in clinical decision-making. Additional technologies, such as frozen section [24] or fluorescence guidance [52], should be examined to increase the nerve-sparing rates of RT-sRP patients to potentially mitigate or ideally eliminate the additional detrimental effect of RT-sRP status on sexual function. Patients with FT-sRP were not at an increased risk of sexual function decline compared to primary RP patients.

Third, within all groups, the impact of RP on the general health-related quality of life (SF-12 physical/mental) was marginal, and decision regret remained low. Similarly, Rodler et al. did not find significant differences in global health scores between 37 RT-sRP and 22 FT-sRP patients [14]. While the assessment and prevalence of decision regret varied in previous reports, the current DRS values were excellent for salvage surgery and were well within the anticipated scope of the primary series [53,54,55,56]. Salvage status was not a significant predictor of HRQOL or decision regret. In contrast, the most commonly used treatment alternative for locally recurrent PCa, namely androgen deprivation therapy (ADT) [1,8,57], was associated with high rates of decision regret in previous reports [58,59,60]. For instance, Wilding et al. reported severe regret in 27.6% of ADT patients, compared to 18.4% of surgically treated patients [58]. Because many men initially opted for FT to preserve erectile and urinary function, it would otherwise be assumed that these men would suffer more from a decline in these domains after RP than primary RP patients due to differences in priorities and attitudes. Therefore, these findings require concomitant consideration in patient counseling when the deterioration of urinary continence or sexual function by RP is discussed and weighted against the potential oncological benefits.

Several limitations must be acknowledged. First, the sample size of sRP patients was small and procedural details of the initial PCa treatment were missing, that is, the RT dose or FT template. Large-scale, multicenter analyses with more granular patient data may be necessary for further subgroup analyses. Not all patients answered all questionnaires every time completely. Second, there is no consensus regarding which PROMs and analytical methods should be used. Psychological factors such as health literacy and the socioeconomic status of the patients were not available for the current analysis. Differences in these factors may have caused systematic bias. Third, the retrospective design of the analysis was prone to selection bias of a high-volume center. Thus, patients referred for salvage RP should not be considered representative of all men who experience local recurrence after RT or FT. However, patients with sRP are generally well selected and sRP should be performed only by highly experienced surgeons. Additionally, the lack of a non-surgical control group limited the comparative assessment of alternative therapies and the value for patient counseling. Fourth, the sexual function domain of the EPIC-26 focuses on erectile function (penetration) and does not cover all biopsychosocial dimensions of sexual health such as desire or psychosocial effects. Fifth, although rigorous multivariable adjustments were universally applied, differences in baseline characteristics between the groups may have artificially amplified the detrimental effects of RT-sRP status.

## 5. Conclusions

Salvage RP is not a uniform procedure, and poor functional outcomes are not inevitable. Our findings indicate that prior FT does not compromise the HRQOL or functional status following RP. Although patients who received RT-sRP had lower urinary continence and sexual function, from an overall PROM perspective, prior treatments did not exert a prohibitive effect that would clearly preclude RP as a treatment option.

## Figures and Tables

**Table 1:** Descriptive characteristics of patients undergoing salvage radical prostatectomy (RP) following prior radiotherapy (RT-sRP) or focal therapy (FT-sRP) compared to primary RP patients between 2014 and 2024 (*n* = 26,515).

	Salvage Radical Prostatectomy		Primary Radical Prostatectomy (n = 26,310, 99.2%)	*p*- Values ^1^
	RT-sRP (*n* = 107, 0.4%)	FT-sRP (*n* = 98, 0.4%)		
Age at RP, in years, median (IQR)	67 (62, 71)	62 (56, 67)	64 (59, 69)	<0.01 <0.01
Months from initial PCa diagnosis to RP, median (IQR)	44 (14, 87)	23 (7, 36)	3 (2, 4)	<0.001 <0.001
Status post transurethral resection of the prostate, *n* (%)	2 (1.9%)	8 (8.2%)	539 (2.0%)	>0.9 <0.001
PSA at RP, in ng/mL, median (IQR)	4.3 (2.3, 7.4)	5.5 (3.0, 8.6)	6.7 (4.5, 10.3)	<0.001 <0.01
Gleason grade group at RP, *n* (%)				<0.001 0.5
1–3	73 (68.2%)	90 (91.8%)	23,640 (89.9%)	
4–5	34 (31.8%)	7 (7.1%)	2526 (9.6%)	
NA	0 (0%)	1 (1.0%)	144 (0.5%)	
pT-stage at RP, *n* (%)				<0.001 0.5
pT2	39 (36.4%)	54 (55.1%)	15,537 (59.1%)	
≥pT3	68 (63.6%)	44 (44.9%)	10,773 (40.9%)	
pN-stage at RP, *n* (%)				<0.001 0.5
pN0/pNX	76 (71.0%)	82 (83.7%)	22,838 (86.8%)	
pN1	31 (29.0%)	16 (16.3%)	3472 (13.2%)	
Nerve-sparing at RP, *n* (%)				<0.001 0.2
Bilaterally	28 (26.2%)	64 (65.3%)	19,364 (73.6%)	
unilaterally	10 (9.3%)	26 (26.5%)	5192 (19.7%)	
no	65 (60.7%)	6 (6.1%)	1733 (6.6%)	
NA	4 (3.7%)	2 (2.0%)	21 (0.1%)	
Margin status at RP, *n* (%)				<0.01 0.3
R0/RX	72 (67.3%)	82 (83.7%)	20,789 (79.0%)	
R1	35 (32.7%)	16 (16.3%)	5521 (21.0%)	
Robot-assisted RP, *n* (%)	14 (13.1%)	40 (40.8%)	15,270 (58.0%)	<0.001 <0.001
Clavien–Dindo > IIIa complications, *n* (%)	18 (16.8%)	7 (7.1%)	1717 (6.5%)	<0.001 >0.9
PROMs available, *n* (%)	95 (88.8%)	92 (93.9%)	23,239 (88.3%)	>0.9 0.1
Years of follow-up, median (IQR)	3 (1, 6)	4 (2, 6)	3 (1, 5)	0.8 0.1

^1^ Differences between sRP and primary RP patients were compared using Wilcoxon rank-sum and Pearson chi-square tests. The first *p*-value refers to the comparison of RT-sRP and primary RP patients. The second *p*-value refers to the comparison of FT-sRP and primary RP patients. Abbreviations: RP = radical prostatectomy, RT-sRP = salvage RP after initial radiotherapy, FT-sRP = salvage RP after initial focal therapy, IQR = interquartile range, NA = not assigned, PROMs = patient-reported outcome measures.

**Table 2:** Patient-reported outcome measures of patients undergoing salvage radical prostatectomy following prior radiotherapy (RT-sRP) or focal therapy (FT-sRP) compared to primary radical prostatectomy patients between 2014 and 2024 (*n* = 26,515).

	RT-sRP		FT-sRP		Primary RP	
	Before RP	After 1 year	Before RP	After 1 year	Before RP	After 1 year
SF-12 physical ^1^	54 (48, 56)	53 (43, 55)	54 (52, 56)	54 (50, 56)	54 (51, 56)	54 (49, 56)
SF-12 mental ^1^	56 (41, 58)	54 (42, 58)	53 (49, 58)	53 (44, 56)	54 (46, 58)	56 (49, 58)
EPIC-26 urinary incontinence ^1^	100 (92, 100)	46 (14, 79) *	100 (92, 100)	86 (58, 100)	100 (94, 100)	92 (67, 100)
EPIC-26 urinary irritation ^1^	88 (75, 100)	88 (75, 94) *	94 (81, 100)	94 (88, 100)	88 (75, 100)	94 (88, 100)
EPIC-26 sexual ^1^	51 (27, 75) *	19 (6, 25) *	72 (56, 94)	29 (19, 48)	75 (54, 88)	33 (19, 54)
EPIC-26 hormonal ^1^	92 (83, 100)	92 (73, 100) *	92 (83, 100)	92 (75, 100) *	92 (83, 100)	100 (83, 100)
EPIC-26 bowel ^1^	100 (94, 100) *	100 (92, 100) *	100 (100, 100)	100 (94, 100)	100 (100, 100)	100 (94, 100)
Full continence ^2^ (no safety pad)	71/74 (95.9%)	15/56 (26.8%) *	88/92 (95.7%)	42/57 (73.7%)	17,504/17,887 (97.9%)	9293/13,750 (67.6%)
Social continence ^2^ (0-1 pad per day)	74/74 (100%)	32/56 (57.1%) *	91/92 (98.9%)	55/57 (96.5%)	17,815/17,887 (99.6%)	12,774/13,750 (92.9 %)
Potency ^2^	22/66 (33.3%) *	1/54 (1.9%) *	62/88 (70.5%)	7/56 (12.5%)	10,703/16,925 (63.2%)	2171/13,480 (16.1%)
Decision Regret Scale ^1^		10 (0, 25)		10 (0, 25)		0 (0, 20)

^1^ median (interquartile range), ^2^ *n* (%). * indicates statistically significant difference compared to reference group (primary RP, *p* < 0.05). Potency was defined as the ability to maintain a spontaneous erection sufficient for penetrative intercourse. Abbreviations: RP = radical prostatectomy, RT-sRP = salvage RP after initial radiotherapy, FT-sRP = salvage RP after initial focal therapy.

## Data Availability

The data generated during the current study are available from the corresponding author on reasonable request.

## References

[1] Tilki D., van den Bergh R.C.N., Briers E., Van den Broeck T., Brunckhorst O., Darraugh J., Eberli D., De Meerleer G., De Santis M., Farolfi A. (2024). EAU-EANM-ESTRO-ESUR-ISUP-SIOG Guidelines on Prostate Cancer. Part II-2024 Update: Treatment of Relapsing and Metastatic Prostate Cancer. Eur. Urol..

[2] Eastham J.A., Auffenberg G.B., Barocas D.A., Chou R., Crispino T., Davis J.W., Eggener S., Horwitz E.M., Kane C.J., Kirkby E. (2022). Clinically Localized Prostate Cancer: AUA/ASTRO Guideline, Part II: Principles of Active Surveillance, Principles of Surgery, and Follow-Up. J. Urol..

[3] National Comprehensive Cancer Network, Inc. (2024). NCCN Guidelines Version 1.2024 Prostate Cancer.

[4] Archer P., Marvaso G., Detti B., Colombo F., Francolini G., Vandendorpe B., Thananayagam M.A., Baty M., De Crevoisier R., Alongi F. (2023). Salvage Stereotactic Reirradiation for Local Recurrence in the Prostatic Bed After Prostatectomy: A Retrospective Multicenter Study. Eur. Urol. Oncol..

[5] Majewski W., Miszczyk M., Graupner D., Goc B., Goldner G., Napieralska A. (2023). Stereotactic Body Radiotherapy (SBRT) Re-Irradiation for Local Failures Following Radical Prostatectomy and Post-Operative Radiotherapy. Strahlenther. Und Onkol..

[6] Crook J.M., Zhang P., Pisansky T.M., Trabulsi E.J., Amin M.B., Bice W., Morton G., Pervez N., Vigneault E., Catton C. (2019). A Prospective Phase 2 Trial of Transperineal Ultrasound-Guided Brachytherapy for Locally Recurrent Prostate Cancer After External Beam Radiation Therapy (NRG Oncology/RTOG-0526). Int. J. Radiat. Oncol. Biol. Phys..

[7] Ambrosini F., Hagemann J., Pose R., Maurer T., Heinzer H., Michl U., Steuber T., Budäus L., Terrone C., Tennstedt P. (2024). Surgical and Oncological Outcomes of Salvage Radical Prostatectomy after Focal Therapies: A Matched-Pair Analysis. World J. Urol..

[8] Chade D.C., Shariat S.F., Cronin A.M., Savage C.J., Karnes R.J., Blute M.L., Briganti A., Montorsi F., van der Poel H.G., Van Poppel H. (2011). Salvage Radical Prostatectomy for Radiation-Recurrent Prostate Cancer: A Multi-Institutional Collaboration. Eur. Urol..

[9] Ogaya-Pinies G., Linares-Espinos E., Hernandez-Cardona E., Jenson C., Cathelineau X., Sanchez-Salas R., Patel V. (2019). Salvage Robotic-Assisted Radical Prostatectomy: Oncologic and Functional Outcomes from Two High-Volume Institutions. World J. Urol..

[10] Bianco F.J., Scardino P.T., Stephenson A.J., Diblasio C.J., Fearn P.A., Eastham J.A. (2005). Long-Term Oncologic Results of Salvage Radical Prostatectomy for Locally Recurrent Prostate Cancer after Radiotherapy. Int. J. Radiat. Oncol. Biol. Phys..

[11] Herrera-Caceres J.O., Nason G.J., Salgado-Sanmamed N., Goldberg H., Woon D.T.S., Chandrasekar T., Ajib K., Tan G.H., Alhunaidi O., van der Kwast T. (2020). Salvage Radical Prostatectomy Following Focal Therapy: Functional and Oncological Outcomes. BJU Int..

[12] Valle L.F., Lehrer E.J., Markovic D., Elashoff D., Levin-Epstein R., Karnes R.J., Reiter R.E., Rettig M., Calais J., Nickols N.G. (2021). A Systematic Review and Meta-Analysis of Local Salvage Therapies After Radiotherapy for Prostate Cancer (MASTER). Eur. Urol..

[13] De Groote R., Nathan A., De Bleser E., Pavan N., Sridhar A., Kelly J., Sooriakumaran P., Briggs T., Nathan S. (2020). Techniques and Outcomes of Salvage Robot-Assisted Radical Prostatectomy (sRARP). Eur. Urol..

[14] Rodler S., Danninger D., Eismann L., Kazmierczak P.M., Jokisch F., Li M., Becker A., Kretschmer A., Stief C., Westhofen T. (2024). Health-Related Quality of Life Following Salvage Radical Prostatectomy for Recurrent Prostate Cancer after Radiotherapy or Focal Therapy. World J. Urol..

[15] Mandel P., Steuber T., Ahyai S., Kriegmair M., Schiffmann J., Boehm K., Heinzer H., Michl U., Schlomm T., Haese A. (2016). Salvage Radical Prostatectomy for Recurrent Prostate Cancer: Verification of European Association of Urology Guideline Criteria. BJU Int..

[16] Gotto G.T., Yunis L.H., Vora K., Eastham J.A., Scardino P.T., Rabbani F. (2010). Impact of Prior Prostate Radiation on Complications after Radical Prostatectomy. J. Urol..

[17] Heidenreich A., Richter S., Thüer D., Pfister D. (2010). Prognostic Parameters, Complications, and Oncologic and Functional Outcome of Salvage Radical Prostatectomy for Locally Recurrent Prostate Cancer after 21st-Century Radiotherapy. Eur. Urol..

[18] Agarwal P.K., Sadetsky N., Konety B.R., Resnick M.I., Carroll P.R. (2008). Cancer of the Prostate Strategic Urological Research Endeavor (CaPSURE) Treatment Failure after Primary and Salvage Therapy for Prostate Cancer: Likelihood, Patterns of Care, and Outcomes. Cancer.

[19] Calleris G., Marra G., Dalmasso E., Falcone M., Karnes R.J., Morlacco A., Oderda M., Sanchez-Salas R., Soria F., Gontero P. (2019). Is It Worth to Perform Salvage Radical Prostatectomy for Radio-Recurrent Prostate Cancer? A Literature Review. World J. Urol..

[20] Ribeiro L., Stonier T., Stroman L., Tourinho-Barbosa R., Alghazo O., Winkler M., Dasgupta P., Popert R., Cathelineau X., Sanchez-Salas R. (2021). Is the Toxicity of Salvage Prostatectomy Related to the Primary Prostate Cancer Therapy Received?. J. Urol..

[21] Onol F.F., Bhat S., Moschovas M., Rogers T., Ganapathi H., Roof S., Rocco B., Patel V. (2020). Comparison of Outcomes of Salvage Robot-Assisted Laparoscopic Prostatectomy for Post-Primary Radiation vs Focal Therapy. BJU Int..

[22] Saouli A., Ruffion A., Dariane C., Barret E., Fiard G., Hankard G.F., Créhange G., Roubaud G., Beauval J.B., Brureau L. (2023). Salvage Radical Prostatectomy for Recurrent Prostate Cancer: A Systematic Review (French ccAFU). Cancers.

[23] Budäus L., Isbarn H., Schlomm T., Heinzer H., Haese A., Steuber T., Salomon G., Huland H., Graefen M. (2009). Current Technique of Open Intrafascial Nerve-Sparing Retropubic Prostatectomy. Eur. Urol..

[24] Ambrosini F., Preisser F., Tilki D., Heinzer H., Salomon G., Michl U., Steuber T., Maurer T., Chun F.K.H., Budäus L. (2024). Nerve-Sparing Radical Prostatectomy Using the Neurovascular Structure-Adjacent Frozen-Section Examination (NeuroSAFE): Results after 20 Years of Experience. Prostate Cancer Prostatic Dis..

[25] Falkenbach F., Mazzucato G., Tian Z., Karakiewicz P.I., Graefen M., Steuber T., Tilki D., Koehler D., Beyer B., Tennstedt P. (2024). Patient-Reported Outcome Measures and Decision Regret After Prostate-Specific Membrane Antigen–Targeted Radioguided Surgery for Oligorecurrent Prostate Cancer. Eur. Urol. Open Sci..

[26] Gandek B., Ware J.E., Aaronson N.K., Apolone G., Bjorner J.B., Brazier J.E., Bullinger M., Kaasa S., Leplege A., Prieto L. (1998). Cross-Validation of Item Selection and Scoring for the SF-12 Health Survey in Nine Countries: Results from the IQOLA Project. International Quality of Life Assessment. J. Clin. Epidemiol..

[27] Ware J., Kosinski M., Keller S.D. (1996). A 12-Item Short-Form Health Survey: Construction of Scales and Preliminary Tests of Reliability and Validity. Med. Care.

[28] Ware J.E., Kosinski M., Keller S.D. (1995). SF-12: How to Score the SF-12 Physical and Mental Health Summary Scales.

[29] Skolarus T.A., Dunn R.L., Sanda M.G., Chang P., Greenfield T.K., Litwin M.S., Wei J.T. (2015). PROSTQA Consortium Minimally Important Difference for the Expanded Prostate Cancer Index Composite Short Form. Urology.

[30] Brehaut J.C., O’Connor A.M., Wood T.J., Hack T.F., Siminoff L., Gordon E., Feldman-Stewart D. (2003). Validation of a Decision Regret Scale. Med. Decis. Mak. Int. J. Soc. Med. Decis. Mak..

[31] O’Connor A.M. (1996). User Manual-Decision Regret Scale [document on the Internet]. Ott. Hosp. Res. Inst..

[32] International Consortium for Health Outcomes Measurement Patient-Centered Outcome Measures Localized Prostate Cancer. https://www.ichom.org/patient-centered-outcome-measure/localized-prostate-cancer/.

[33] Wei J.T., Dunn R.L., Litwin M.S., Sandler H.M., Sanda M.G. (2000). Development and Validation of the Expanded Prostate Cancer Index Composite (EPIC) for Comprehensive Assessment of Health-Related Quality of Life in Men with Prostate Cancer. Urology.

[34] Beyer B., Huland H., Feick G., Graefen M. (2015). “Expanded prostate cancer index composite” (EPIC-26): Results of functional treatment in patients with localized prostate cancer. Der Urol..

[35] Umbehr M.H., Bachmann L.M., Poyet C., Hammerer P., Steurer J., Puhan M.A., Frei A. (2018). The German Version of the Expanded Prostate Cancer Index Composite (EPIC): Translation, Validation and Minimal Important Difference Estimation. Health Qual. Life Outcomes.

[36] Clavien P.A., Barkun J., de Oliveira M.L., Vauthey J.N., Dindo D., Schulick R.D., de Santibanes E., Pekolj J., Slankamenac K., Bassi C. (2009). The Clavien-Dindo Classification of Surgical Complications: Five-Year Experience. Ann. Surg..

[37] R Core Team (2023). R: A Language and Environment for Statistical Computing.

[38] Kaffenberger S.D., Keegan K.A., Bansal N.K., Morgan T.M., Tang D.H., Barocas D.A., Penson D.F., Davis R., Clark P.E., Chang S.S. (2013). Salvage Robotic Assisted Laparoscopic Radical Prostatectomy: A Single Institution, 5-Year Experience. J. Urol..

[39] Kenney P.A., Nawaf C.B., Mustafa M., Wen S., Wszolek M.F., Pettaway C.A., Ward J.F., Davis J.W., Pisters L.L. (2016). Robotic-Assisted Laparoscopic versus Open Salvage Radical Prostatectomy Following Radiotherapy. Can. J. Urol..

[40] Yuh B., Ruel N., Muldrew S., Mejia R., Novara G., Kawachi M., Wilson T. (2014). Complications and Outcomes of Salvage Robot-Assisted Radical Prostatectomy: A Single-Institution Experience. BJU Int..

[41] Marconi L., Stonier T., Tourinho-Barbosa R., Moore C., Ahmed H.U., Cathelineau X., Emberton M., Sanchez-Salas R., Cathcart P. (2019). Robot-Assisted Radical Prostatectomy After Focal Therapy: Oncological, Functional Outcomes and Predictors of Recurrence. Eur. Urol..

[42] Nunes-Silva I., Barret E., Srougi V., Baghdadi M., Capogrosso P., Garcia-Barreras S., Kanso S., Tourinho-Barbosa R., Carneiro A., Sanchez-Salas R. (2017). Effect of Prior Focal Therapy on Perioperative, Oncologic and Functional Outcomes of Salvage Robotic Assisted Radical Prostatectomy. J. Urol..

[43] Marra G., Gontero P., Walz J.C., Sivaraman A., Tourinho-Barbosa R., Cathelineau X., Sanchez-Salas R. (2019). Complications, Oncological and Functional Outcomes of Salvage Treatment Options Following Focal Therapy for Localized Prostate Cancer: A Systematic Review and a Comprehensive Narrative Review. World J. Urol..

[44] Zargar H., Lamb A.D., Rocco B., Porpiglia F., Liatsikos E., Davis J., Coelho R.F., Pow-Sang J.M., Patel V.R., Murphy D.G. (2017). Salvage Robotic Prostatectomy for Radio Recurrent Prostate Cancer: Technical Challenges and Outcome Analysis. Minerva Urol. Nefrol. Ital. J. Urol. Nephrol..

[45] Hudak S.J., Morey A.F. (2011). Impact of 3.5 Cm Artificial Urinary Sphincter Cuff on Primary and Revision Surgery for Male Stress Urinary Incontinence. J. Urol..

[46] Short-, Intermediate-, and Long-Term Quality of Life Outcomes Following Radical Prostatectomy for Clinically Localized Prostate Cancer. https://pubmed.ncbi.nlm.nih.gov/24659913/.

[47] Donovan J.L., Hamdy F.C., Lane J.A., Mason M., Metcalfe C., Walsh E., Blazeby J.M., Peters T.J., Holding P., Bonnington S. (2016). Patient-Reported Outcomes after Monitoring, Surgery, or Radiotherapy for Prostate Cancer. N. Engl. J. Med..

[48] Donovan J.L., Hamdy F.C., Lane J.A., Young G.J., Metcalfe C., Walsh E.I., Davis M., Steuart-Feilding T., Blazeby J.M., Avery K.N.L. (2023). Patient-Reported Outcomes 12 Years after Localized Prostate Cancer Treatment. NEJM Evid..

[49] Chen R.C., Basak R., Meyer A.-M., Kuo T.-M., Carpenter W.R., Agans R.P., Broughman J.R., Reeve B.B., Nielsen M.E., Usinger D.S. (2017). Association Between Choice of Radical Prostatectomy, External Beam Radiotherapy, Brachytherapy, or Active Surveillance and Patient-Reported Quality of Life Among Men With Localized Prostate Cancer. JAMA.

[50] Hoffman K.E., Penson D.F., Zhao Z., Huang L.-C., Conwill R., Laviana A.A., Joyce D.D., Luckenbaugh A.N., Goodman M., Hamilton A.S. (2020). Patient-Reported Outcomes Through 5 Years for Active Surveillance, Surgery, Brachytherapy, or External Beam Radiation With or Without Androgen Deprivation Therapy for Localized Prostate Cancer. JAMA.

[51] Lane J.A., Donovan J.L., Young G.J., Davis M., Walsh E.I., Avery K.N.L., Blazeby J.M., Mason M.D., Martin R.M., Peters T.J. (2022). Functional and Quality of Life Outcomes of Localised Prostate Cancer Treatments (Prostate Testing for Cancer and Treatment [ProtecT] Study). BJU Int..

[52] Nguyen H.G., van den Berg N.S., Antaris A.L., Xue L., Greenberg S., Rosenthal J.W., Muchnik A., Klaassen A., Simko J.P., Dutta S. (2023). First-in-Human Evaluation of a Prostate-Specific Membrane Antigen–Targeted Near-Infrared Fluorescent Small Molecule for Fluorescence-Based Identification of Prostate Cancer in Patients with High-Risk Prostate Cancer Undergoing Robotic-Assisted Prostatectomy. Eur. Urol. Oncol..

[53] Lunger L., Meissner V.H., Kopp B.C.G., Dinkel A., Schiele S., Ankerst D.P., Gschwend J.E., Herkommer K. (2023). Prevalence and Determinants of Decision Regret in Long-Term Prostate Cancer Survivors Following Radical Prostatectomy. BMC Urol..

[54] Gartrell B.A., Phalguni A., Bajko P., Mundle S.D., McCarthy S.A., Brookman-May S.D., De Solda F., Jain R., Yu Ko W., Ploussard G. (2024). Influential Factors Impacting Treatment Decision-Making and Decision Regret in Patients with Localized or Locally Advanced Prostate Cancer: A Systematic Literature Review. Eur. Urol. Oncol..

[55] Meissner V.H., Simson B.W., Dinkel A., Schiele S., Ankerst D.P., Lunger L., Gschwend J.E., Herkommer K. (2023). Treatment Decision Regret in Long-Term Survivors after Radical Prostatectomy: A Longitudinal Study. BJU Int..

[56] Wolff I., Burchardt M., Peter J., Thomas C., Sikic D., Fiebig C., Promnitz S., Hoschke B., Burger M., Schnabel M.J. (2023). Patient’s Desire and Real Availability Concerning Supportive Measures Accompanying Radical Prostatectomy: Differences between Certified Prostate Cancer Centers and Non-Certified Centers Based on Patient-Reported Outcomes within the Cross-Sectional Study Improve. Cancers.

[57] Cary K.C., Paciorek A., Fuldeore M.J., Carroll P.R., Cooperberg M.R. (2014). Temporal Trends and Predictors of Salvage Cancer Treatment after Failure Following Radical Prostatectomy or Radiation Therapy: An Analysis from the CaPSURE Registry. Cancer.

[58] Wilding S., Downing A., Selby P., Cross W., Wright P., Watson E.K., Wagland R., Kind P., Donnelly D.W., Hounsome L. (2020). Decision Regret in Men Living with and beyond Nonmetastatic Prostate Cancer in the United Kingdom: A Population-Based Patient-Reported Outcome Study. Psychooncology.

[59] Morris B.B., Farnan L., Song L., Addington E.L., Chen R.C., Nielsen M.E., Mishel M., Mohler J.L., Bensen J.T. (2015). Treatment Decisional Regret among Men with Prostate Cancer: Racial Differences and Influential Factors in the North Carolina Health Access and Prostate Cancer Treatment Project (HCaP-NC). Cancer.

[60] Nguyen P.L., Alibhai S.M., Basaria S., D’Amico A.V., Kantoff P.W., Keating N.L., Penson D.F., Rosario D.J., Tombal B., Smith M.R. (2015). Adverse Effects of Androgen Deprivation Therapy and Strategies to Mitigate Them. Eur. Urol..

